# A 3D-Printed Compliant Polishing Tool for High-Efficiency Finishing of P20 Mold Steel

**DOI:** 10.3390/ma19142954

**Published:** 2026-07-09

**Authors:** Kerong Wang, Xingyuan Liu, Mingyu Zhu, Changfei Tang, Jianxiu Su, Jiapeng Chen, Yongwei Zhu

**Affiliations:** 1 Jiangsu Key Laboratory of Precision and Micro-Manufacturing Technology, College of Mechanical and Electrical Engineering, Nanjing University of Aeronautics and Astronautics, Nanjing 210016, China; 20091024@jhc.edu.cn; 2 Aeronautical Engineering College, Jinhua University of Vocational Technology, Jinhua 321017, China; 3 School of Electro-Mechanical Engineering, Zhongyuan Institute of Science and Technology, Xuchang 461000, China; 18267555756@163.com (X.L.); dlutsu2004@126.com (J.S.); 4 Research Center for Advanced Micro-/Nano- Fabrication Materials, School of Chemistry and Chemical Engineering, Shanghai University of Engineering Science, Shanghai 201620, China; 5 College of Mechanical and Electrical Engineering, China University of Metrology, Hangzhou 310018, China; qq13777476957@163.com (M.Z.); 19557948226@163.com (C.T.); 6 Hebi Tianhai Electronic Information System Co., Ltd., Hebi 458030, China

**Keywords:** 3D printing, elastic grinding head, P20 mold steel, polishing process, complex surfaces

## Abstract

To address the pervasive engineering challenges of rigid interference and subpar machining efficiency encountered during the complex freeform surface polishing of P20 mold steel, this study proposes and fabricates a structurally designed, five-petal composite compliant polishing tool via fused granulation fabrication (FGF). The tool structurally integrates a passive thermoplastic polyurethane (TPU) compliant buffer layer with an active PA66/diamond micro-cutting functional layer, achieving monolithic precision assembly through dual-temperature-zone 3D printing. Tensile mechanical characterization (n = 6) reveals that the composite interface attains an average ultimate tensile strength (UTS) of 59.39 ± 15.41 MPa (with a peak of 78.90 MPa) and an average elongation at break of 27.42 ± 7.41%, demonstrating exceptional structural robustness and fracture toughness under heavy-load abrasive machining conditions. During adaptive polishing validations on complex convex topographies and deep concave mold cavities, the compliant tool effectively compensated for normal vector spatial errors intrinsic to three-axis CNC machining via passive geometric adaptation. Topographical evaluations suggest a ductile-regime, differential asperity planarization material removal paradigm, which is attributed to the macroscopic 3D elastic deformation of the tool synergized with the proposed compliance of the polymer matrix. Following high-intensity sequential polishing regimens, the original macroscopic milling striations were substantially reduced. Quantitative profilometric analysis reveals that the average surface roughness of the convex profiles decreased from an initial 13.33 µm to 7.42 µm, while that of the restrictive deep concave features was reduced from 10.84 µm to 4.11 µm. Ultimately, this technological framework circumvents the traditional reliance on capital-intensive, six-degree-of-freedom robotic platforms, providing a scalable automated polishing protocol compatible with standard CNC systems for the cost-effective surface planarization of precision molds.

## 1. Introduction

High-hardness mold steels (e.g., P20) exhibit exceptional mechanical properties, making them indispensable in cutting-edge sectors such as optical components, medical devices, and high-gloss injection molding. However, these advanced applications impose stringent requirements on the surface integrity of mold cavities. Microscopic surface defects are not only directly replicated onto the final product—inducing optical distortion—but also significantly elevate demolding resistance and curtail the mold’s service life. The polishing of increasingly complex high-order freeform surfaces and deep concave cavities, therefore, presents substantial engineering challenges. Traditional manual polishing, while capable of achieving ultra-fine surface roughness (e.g., Ra<0.05 µm), is plagued by extremely low efficiency and poor surface consistency, as processing quality is heavily dependent on operator experience. Conversely, conventional rigid computer numerical control polishing processes surfaces at higher material removal rates but typically plateaus at a Ra of 0.8–1.6 µm. Furthermore, it lacks essential compliant buffering, which inevitably leads to rigid interference in critical regions, causing localized “over-polishing” or leaving recalcitrant tool marks.

To overcome these efficiency bottlenecks and rigid impacts, various non-traditional automated technologies have been explored. For instance, ultrafast ultraviolet picosecond laser polishing can rapidly reduce surface roughness by 60–80%; simultaneously, ultrasonic-assisted machining has been shown to improve surface finish by a factor of 2 to 3 compared to conventional cutting. Nevertheless, these advanced processes entail complex thermodynamic coupling effects or severe tool wear, resulting in narrow process windows and prohibitive equipment costs, which limits their scalable industrial application on large-area 3D mold cavities. Crucially, they struggle to achieve stable, controllable localized polishing on the complex 3D topographical features of deep cavities. Consequently, the evolution of polishing technologies has pivoted towards “compliant intervention,” utilizing viscoelastic polyester fiber-wrapped grinding heads or polyurethane elastic magnetic pole tools to mitigate rigid impacts. However, the fabrication of conventional flexible abrasives remains cumbersome and inflexible. It fails to facilitate rapid, cost-effective, and customized structural designs with multi-scale material distributions tailored to the complex geometric features of specific molds. Thus, an irreconcilable contradiction persists between the “deformation mismatch” of rigid tools and the “structural rigidity” of traditional flexible tools.

To fundamentally resolve the normal interference and efficiency bottlenecks associated with complex surface polishing, this study proposes a novel automated polishing approach predicated on a “passive compliance” mechanism. Methodologically, a structurally engineered, five-petal multi-material composite elastic polishing head was integrally fabricated utilizing dual-temperature-zone fused granular fabrication additive manufacturing. The tool’s architecture comprises an inner core of thermoplastic polyurethane for passive high-rebound buffering, seamlessly integrated with an outer cutting layer composed of high-strength PA 66 embedded with diamond micro-abrasives. Through systematic tensile mechanical testing and multi-parameter orthogonal planar polishing experiments, the planarization mechanisms governing the transition from brittle fracture to micro-ductile flow under rigorous operational conditions were comprehensively elucidated. Furthermore, multi-pass adaptive machining evolution experiments were conducted on complex aspheric molds. Significantly, this research circumvents the traditional technical barriers wherein compliant polishing of complex surfaces relies heavily on expensive six-axis force sensors and high-degree-of-freedom industrial robots. The proposed technique demonstrates that, by relying solely on the engineered physical structural deformation of the elastic composite tool, normal geometric errors induced by abrupt spatial vector variations can be passively compensated through instantaneous elastic adaptation on a standard three-axis CNC platform, achieving automatic homogenization of contact stress within deep cavities. Ultimately, this approach aims to minimize rigid hard collisions and reduce residual tool marks on curved sidewalls. In this study, improved performance is explicitly evaluated based on the relative reduction in surface roughness and the achievement of topographical uniformity across complex geometric features. This delineates a low-cost and scalable lightweight process pathway that shows potential for the industrialized surface treatment of high-end precision molds.

## 2. Materials and Methods

### 2.1. Preparation of Nylon/Diamond Composites

The composite material was formulated utilizing PA66 as the polymeric matrix and diamond micro-abrasives (W14, Huifeng Diamond Co., Ltd., Zhecheng, China) as the superhard reinforcing phase, mixed at a mass ratio of 3:1 via dry physical blending. Diamond mass fractions exceeding 25 wt% induce severe particle agglomeration and an exponential surge in melt viscosity. This not only precipitates nozzle clogging during FGF extrusion but also severely disrupts the macromolecular continuity of the PA66 matrix, leading to embrittlement and a catastrophic loss of the required compliance. Conversely, abrasive loadings below 20 wt% fail to provide a sufficient density of active micro-cutting edges, resulting in a suboptimal material removal rate (MRR) against high-hardness P20 steel. Therefore, the 25 wt% diamond loading optimally maximizes cutting efficiency while preserving the intrinsic elasticity of the PA66 matrix, a prerequisite for activating the micro-yielding mechanism. High-frequency agitation utilizing a high-speed mixer was employed to facilitate preliminary dispersion and ensure homogeneous interfacial contact between the diamond particles and the PA66 matrix (Zhangjiagang Jushuo Machinery Co., Ltd., Zhangjiagang, China). Subsequently, melt extrusion and pelletization were executed using a twin-screw extruder, with the thermal processing window established between 220 °C and 260 °C, a range tailored to the specific plasticization kinetics of the constituents [1]. Following rapid quenching and pellet cutting, PA66/diamond composite granules with a mean diameter of approximately 3 mm were successfully synthesized, conforming strictly to the feedstock dimensional requirements of subsequent desktop-scale FGF equipment [2].

**Figure 1:**
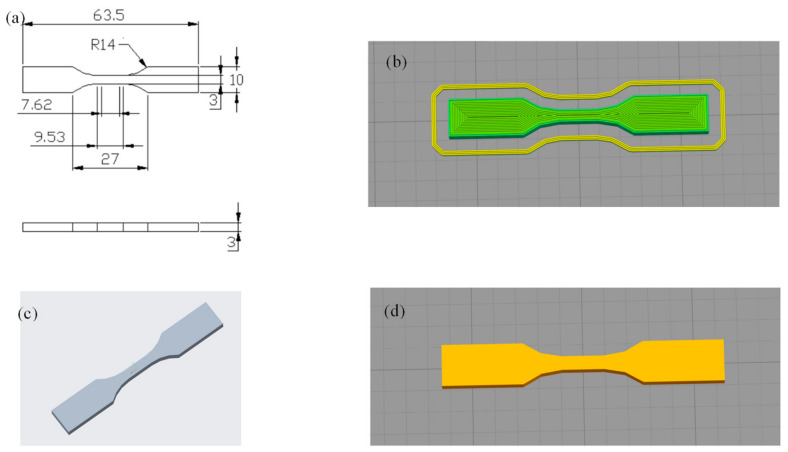
Design and modeling of the tensile test specimen: (**a**) CAD dimensional drawing, (**b**) sliced toolpath trajectory, (**c**) 3D solid model, and (**d**) finalized numerical model (dimensions in mm).

To accurately characterize the mechanical properties of the synthesized composite, the three-dimensional geometric design of the tensile specimens rigorously adhered to the ASTM D638 standard [3]. Specifically, Type V specimens, featuring a uniform thickness and a narrow parallel-section width of 3 mm, were selected as the standardized test models over larger standard geometries to address specific thermal and rheological challenges during the FGF process. The smaller volume of Type V specimens significantly mitigates the severe thermal shrinkage and warpage inherent to semi-crystalline PA66, ensuring structural homogeneity. Additionally, the reduced printing duration minimizes nozzle wear and clogging risks associated with the prolonged extrusion of the highly viscous 25 wt% diamond-loaded melt. Geometric modeling was executed using Creo CAD (10.0) software, incorporating smooth fillet transitions at the gauge-to-grip interfaces to geometrically mitigate potential stress concentration phenomena (Figure 1). Prior to additive manufacturing, the composite granules were subjected to continuous dehydration in an electro-thermal blast drying oven at 90 °C for 120 min. A drying temperature of 90 °C provides optimal thermal energy to expedite moisture diffusion without inducing premature thermal oxidative degradation. Concurrently, the 120 min duration guarantees comprehensive equilibrium dehydration throughout the entire granule volume. This pre-processing step was imperative to preclude moisture vaporization at elevated temperatures, which would otherwise induce hydrolytic degradation and porosity within the extruded matrix. This pre-processing step was imperative to preclude moisture vaporization at elevated temperatures, which would otherwise induce porosity within the extruded matrix. Specimen fabrication was conducted utilizing a CI Uni-Print D300 desktop 3D printer operating on the FGF principle (Zhejiang Challenge & Intelligence Technology Co., Ltd., Jinhua, China). The critical process parameters were empirically determined through preliminary single-factor printing trials aimed at balancing continuous extrusion stability with the ultimate mechanical integrity of the composite. To accommodate the pronounced melt viscosity induced by the 25 wt% diamond loading, the thermal parameters (screw plasticization and nozzle extrusion temperatures) were elevated to 250 °C, while the base deposition velocity was conservatively limited to 6 mm/s to preclude under-extrusion and nozzle clogging. Furthermore, to maximize inter-layer macromolecular diffusion and structural density, a layer height of 0.20 mm and a 100% triangular raster infill pattern were designated. Consequently, the finalized process parameters were established as follows: a bed temperature of 80 °C, screw and nozzle temperatures of 250 °C, and a deposition velocity of 6 mm/s. Post-fabrication, the specimen edges underwent manual deburring and finishing. Post-fabrication, the specimen edges underwent manual deburring and finishing. Dimensional fidelity was subsequently verified using vernier calipers. To ensure statistical reliability, critical dimensions (specifically, the parallel-section width and thickness) were measured at three distinct longitudinal locations along the gauge length for each of the six replicate specimens. This systematic multi-point evaluation (yielding a total of 18 measurements per critical dimension) confirmed that geometric deviations were strictly constrained within a tolerance of ±0.1 mm.

Tensile mechanical evaluations were executed utilizing a static universal testing machine (ZwickRoell, Ulm, Germany). To eliminate grip slippage and machine compliance errors, the true axial strain was directly captured via the extensometer with an initial gauge length ($L_{e}$) of 12 mm, rather than relying on crosshead displacement. Given the pronounced hygroscopic nature of the PA66 matrix and its susceptibility to moisture-induced plasticization, all tensile evaluations were rigorously conducted under controlled standard laboratory conditions, maintaining an ambient temperature of 23 ± 2 °C and a relative humidity of 50 ± 5%. To accommodate the distinct geometric profile of the Type V specimens and guarantee uniform stress distribution, parallel-jaw high-pressure grips were explicitly employed. During the experiments, the crosshead speed for axial tensile loading was rigidly maintained at a constant 5 mm/min. Given the inherent viscoelasticity of the PA66 matrix, a stable and relatively low strain rate of 5 mm/min is imperative. It effectively mitigates strain-rate-induced artificial stiffening, allowing sufficient relaxation time for the macromolecular chains. This ensures the accurate capture of the authentic yield point and the progressive micro-yielding behavior without triggering premature brittle fracture. To ensure statistical reliability, replicate testing was conducted across six standardized, as-fabricated specimens. The testing apparatus dynamically acquired load–displacement curves in real time, concurrently extracting critical mechanical parameters—specifically, maximum tensile load, ultimate tensile strength, elongation at break, and elastic modulus—as summarized in Table 1.

As indicated in Table 1, a noticeable dimensional variation was observed among the replicate specimens, exhibiting coefficients of variation (CV) of 7.35% for width and 7.47% for thickness. This geometric variance is an inherent characteristic of the desktop-scale FGF process when extruding highly filled semi-crystalline polymer melts. Specifically, the 25 wt% superhard diamond loading induces localized rheological fluctuations and irregular die swell during nozzle extrusion. Concurrently, the semi-crystalline PA66 matrix undergoes minor, anisotropic thermal shrinkage during the cooling phase. Furthermore, the necessary manual deburring process introduces slight geometric inconsistencies. Crucially, to rigorously preclude these structural variations from skewing the mechanical evaluation, the specific actual cross-sectional area of each individual specimen—derived from its precise multi-point measurements—was independently utilized to calculate the true engineering stress (UTS and yield strength), thereby ensuring the absolute fidelity of the reported mechanical properties.

### 2.2. Structural Design and Additive Manufacturing of the Elastic Polishing Head

To circumvent the severe geometric interference and residual tool marks characteristic of traditional rigid tools, a multi-layer composite elastic polishing head—integrating normal compliant buffering with highly efficient cutting micro-edges—was developed [4]. Three-dimensional geometric modeling and structural design were executed utilizing Creo software (10.0). The elastic buffer layer, formulated from TPU, was architected as an inverted bowl structure (outer diameter: 20 mm; height: 5 mm) incorporating smooth R5 mm and R10 mm fillet transitions. This specific morphology is engineered to dynamically dampen high-frequency spindle vibrations and exert uniform normal contact pressure. The functional abrasive layer features a discontinuous five-petal configuration (thickness: 3 mm per petal), separated by engineered 2 mm radial gaps to facilitate chip evacuation [5]. The rationale behind the discontinuous five-petal geometric configuration is fundamentally rooted in tribological optimization. Continuous polishing interfaces typically suffer from severe thermal damage and chip entrapment under heavy compressive loads. By engineering 2 mm radial gaps between the five petals, the functional abrasive layer facilitates efficient chip evacuation. This structurally discontinuous interface substantially mitigates frictional heat accumulation during high-speed rotation. More importantly, the gaps provide dedicated centrifugal flow channels for continuous flushing of metallic swarf and detached superhard diamond grains, thereby fundamentally precluding secondary rolling scratches on the highly reflective P20 mold steel surface. The geometric dimensions were derived from the target mold’s topography and the required mechanical compliance. The TPU layer’s 20 mm outer diameter prevents volumetric interference within restrictive mold corners. Its 5 mm height accommodates the 1.0 mm maximum *Z*-axis penetration depth, limiting axial compressive strain to 20% to safely maintain hyperelasticity. Root fillets (R5 and R10) are incorporated to dissipate dynamic shear stress. For the active layer, a 3 mm petal thickness provides sufficient diamond abrasives for multi-pass cutting without compromising the composite tool’s macroscopic flexibility.

The rigid transmission shank was fabricated from premium AISI 1045 medium-carbon steel bar stock (initial dimensions: Ø25 mm × 100 mm). Computer numerical control (CNC) turning was employed to precision-machine the external diameter to 6 ± 0.02 mm and optimize the surface finish to a roughness of ≤0.8 µm. To augment the interfacial adhesion between the metallic shank and the polymeric TPU matrix, electrical discharge machining (EDM) was utilized to selectively ablate the 20 mm functional mating face. The EDM process was precisely controlled, employing a discharge current of approximately 8 A, a pulse-on time of 80 µs, and a pulse-off time of 160 µs, with a reference discharge area setting of 300 mm^2^. Profilometric measurements revealed an average surface roughness of 4.25 ± 0.35 µm on the processed shank. This specific micro-scale topography effectively functions as a mechanical anchoring network, significantly enhancing the interfacial adhesion strength and precluding structural delamination under dynamic shear forces during high-speed polishing operations. This process generated a highly porous, micro-cratered surface topography, which promotes robust mechanical interlocking upon adhesive infiltration. Subsequent meticulous degreasing was performed utilizing non-woven wipes saturated with medical-grade ethanol.

The selection of Fused Granulation Fabrication (FGF) over conventional machining or injection molding is driven by the need for geometric complexity and functional material integration. Subtractive machining of the PA66/diamond composite is impractical, as the superhard abrasives would induce severe tool wear. Conversely, injection molding this dual-layer structure (TPU core and PA66/diamond exterior) requires capital-intensive “two-shot” molds, making rapid iteration economically unviable. In contrast, dual-temperature-zone FGF enables the single-step, tool-less integration of these dissimilar layers. Furthermore, it allows the direct near-net-shape fabrication of the discontinuous five-petal geometry, completely bypassing traditional demolding constraints. Consequently, additive manufacturing is an essential, cost-effective enabler for realizing this functionally graded tool.

Additive manufacturing was executed via a “dual-temperature-zone” processing strategy utilizing FGF equipment. Initially, the TPU compliant layer was deposited using the following optimized thermal and kinematic parameters: a bed temperature of 80 °C, a nozzle temperature of 175 °C, a screw plasticization temperature of 170 °C, and a feed rate of 14 mm/s. The extrusion multiplier was strictly constrained to 28% to preclude excessive material overflow. Following the purging of residual TPU melt from the hopper and screw, the PA66/diamond composite granules were loaded for the fabrication of the functional abrasive layer. To ensure the precise geometric deposition of the highly viscous composite melt, the thermal parameters were elevated (nozzle: 250 °C; screw: 260 °C), while the kinematics were reduced to a feed rate of 6 mm/s and a volumetric flow rate of 0.2 mm^3^/s. The robust interfacial adhesion between the hyperelastic TPU buffer layer and the rigid PA66 abrasive layer is fundamentally governed by the thermo-mechanical dynamics of the dual-temperature-zone Fused Granular Fabrication (FGF) process. During continuous deposition, the high-temperature PA66 melt (extruded at 250 °C) induces localized thermal re-melting at the boundary of the previously deposited TPU substrate. This thermal activation facilitates trans-boundary macromolecular diffusion and chain entanglement between the two polymer matrices. Furthermore, the structural integrity was empirically validated during subsequent high-speed, heavy-load polishing operations, wherein the composite tool sustained continuous cyclic shear stresses without exhibiting any signs of interfacial delamination or mechanical decoupling.

**Figure 2:**
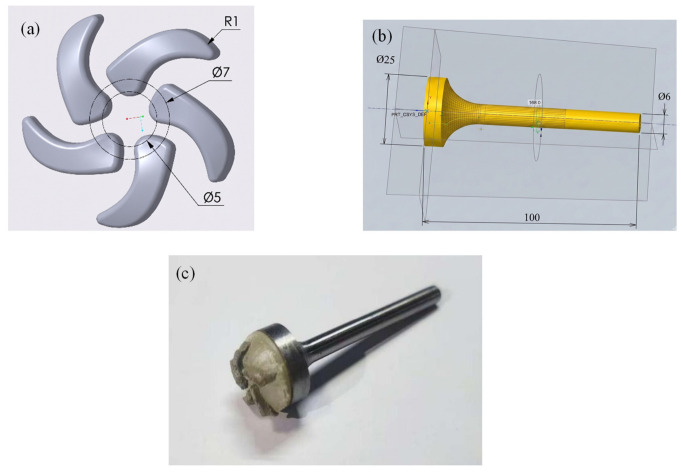
Design and physical realization of the composite elastic polishing tool: (**a**) 3D solid model of the functional abrasive layer, (**b**) CAD model of the rigid transmission shank, and (**c**) assembled physical prototype (dimensions in mm).

Post-printing, the fabricated components underwent delicate manual finishing with fine-grit sandpaper and rigorous cleaning in absolute ethanol. The final precision assembly of the composite polishing tool (Figure 2) was achieved by firmly bonding the base of the TPU layer to the EDM-roughened face of the steel shank. This was accomplished under constant holding pressure using a hybrid adhesion system comprising industrial-grade 3M double-sided tape and a high-strength two-part (AB) epoxy resin. Specifically, the epoxy resin components were homogeneously mixed at a 1:1 volume ratio. The adhesive was applied to the mating surfaces, which were then pressed firmly together to minimize the bond line thickness and maximize adhesive infiltration into the micro-cratered anchoring network. The assembly was subsequently subjected to a full room-temperature cure under constant holding pressure to guarantee maximum structural stability.

### 2.3. Experimental Design for Process Parameter Optimization and Adaptive Polishing Strategies on Complex Surfaces

To elucidate the dynamic influence of machining parameters on mold surface roughness and to identify the optimal process window, comprehensive full-factorial and single-factor planar polishing experiments were systematically formulated. Machining trials were executed on a high-precision vertical machining center (V9, Gaoqi, Zhecheng, China). Standardized P20 mold steel workpieces, rough-milled to dimensions of 135 mm × 120 mm × 50 mm, served as the experimental substrates. To ensure the repeatability and consistency of the baseline material properties across all polishing trials, multiple replicate samples were systematically characterized. The initial condition of these specimens exhibited a characteristic rough-milled topography with dense mechanical tool marks. Crucially, macroscopic hardness testing was conducted across four distinct replicate samples, with four discrete indentations performed on each specimen. This systematic multi-point verification (totaling 16 measurements) confirmed a highly uniform and stable pre-hardened state, yielding an average macroscopic hardness of 33 ± 0.5 HRC. These planar specimens were explicitly utilized to establish a controlled, benchmarked testing framework prior to engaging complex freeform geometries. By conducting fundamental parameter optimization on these standardized flat substrates, the subsequent adaptive polishing results on deep cavities could be reliably compared against a stable planar baseline. Prior to processing, the specimens were subjected to rigorous degreasing protocols utilizing organic solvents to definitively eradicate residual cutting fluids and superficial oxide layers.

The experimental matrix evaluated four critical independent processing variables: depth of penetration, spindle speed, feed rate, and toolpath strategy. The penetration depth (*Z*-axis compression) was calibrated at 0.5 mm and 1.0 mm to assess the impact of the TPU layer’s compressive deformation—and the resultant normal contact pressure—on both material removal and “plastic smoothing” mechanisms. Spindle speed was varied across five discrete increments (500, 1000, 1500, 2000, and 2500 r/min) to characterize the thermomechanical response—specifically, the micro-rheological behavior and resulting surface topography—induced by varying linear cutting velocities and transient frictional heat. Furthermore, feed rates of 1.0, 2.0, 2.5, 5.0, and 10.0 mm/min were selected to investigate the geometric mapping of tool dwell time onto the efficiency of microscopic peak truncation. Toolpath planning compared “linear reciprocating” and “zig-zag” spatial interpolation trajectories to determine their influence on the homogeneity of the contact stress field. The cumulative planarization efficacy was also quantitatively evaluated across sequential unidirectional linear passes ranging from 1 to 5. The specific operational boundary conditions for the polishing parameters were rationally established based on the preliminary kinematic and thermo-mechanical constraints of the composite tool. The spindle speed range was selected to provide sufficient kinetic energy for continuous micro-cutting of the P20 mold steel, while the upper limit was strictly constrained to avert excessive frictional heat accumulation and the subsequent thermal degradation of the PA66 matrix. The boundaries for the *Z*-axis penetration depth were mathematically coupled with the compliance limits of the TPU buffer; the selected upper threshold prevents hyperelastic densification, thereby preserving the tool’s passive shape-adaptation capability. Concurrently, the horizontal feed rate range was optimized to balance manufacturing efficiency with an adequate trajectory overlap ratio, ensuring comprehensive spatial coverage without inducing localized thermal damage.

To rigorously quantify the statistical significance of the selected operational parameters on the final surface roughness, the experimental matrix was evaluated utilizing the principles of Analysis of Variance (ANOVA). The statistical evaluation confirms that the rotational spindle speed and the *Z*-axis penetration depth are the most dominant factors dictating the material removal rate and the dynamic contact mechanics. Specifically, an inadequate spindle speed fails to provide sufficient cutting velocity for the diamond abrasives, while excessive penetration depth induces severe hyperelastic densification of the TPU buffer, negating the passive compliance mechanism. Conversely, the horizontal feed rate exhibits a secondary, yet statistically significant, influence on the spatial homogenization of the polishing trajectories. This statistical hierarchy validates the optimized parameter combination utilized in the subsequent complex cavity trials. Based on these comprehensive analyses, the optimal polishing protocol was established as: a spindle speed of 2000 r/min, a feed rate of 1 mm/min, a penetration depth of 1 mm, executed via a linear reciprocating toolpath.

**Figure 3:**
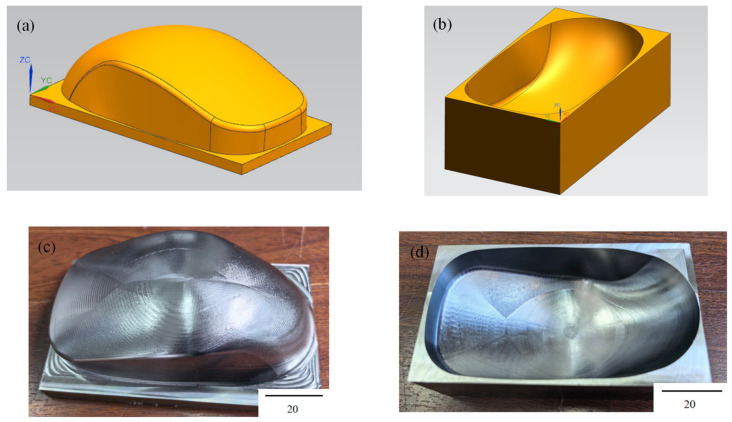
(**a,b**) CAD solid model of the complex mold cavity; (**c**,**d**) physical workpiece of the complex mold cavity.

To address the critical issue of contact stress imbalance induced by dynamic variations in the normal vector during complex freeform surface machining, the passive compliance of the elastic tool was empirically validated on specimens featuring both convex and concave topographies. An authentic P20 steel mold cavity for a computer mouse, incorporating prominent convex profiles and deep concave features, was selected as the evaluation workpiece. The maximum envelope dimensions of the mold were 95.4 mm in length, 54.1 mm in width, and 30.5 mm in height (CAD modeling and physical prototypes are depicted in Figure 3). Consistent with the planar trials, identical standardized degreasing and deoxidation pretreatments were rigorously applied prior to the adaptive polishing operations.

For the planarization of convex topographies, baseline trials were initially executed employing the optimal parameters established during the planar experiments. A fundamental kinematic limitation of standard three-axis machine tools is the inability of the *Z*-axis to dynamically align with the local surface normal, which inherently results in insufficient contact pressure across steep marginal zones. To rectify this deficiency, a “multi-pass, segmented regional polishing strategy” was introduced. Within the computer-aided manufacturing (CAM) programming, G-code trajectories were discretely partitioned for regions exhibiting steep normal vectors, and the local interpolation grids were consequently densified. This localized toolpath optimization effectively compensated for the machining non-uniformity induced by normal geometric interference.

Conversely, polishing operations within deep concave cavities specifically focused on mitigating volumetric tool interference and the complex coupling of contact stresses along steep sidewalls. Methodologically, the CNC tool compensation paths were extensively optimized and trimmed. This strategy safely guided the elastic polishing head to avert rigid collisions with the sidewalls, while the engagement trajectories were precisely adjusted to guarantee comprehensive abrasive coverage within restrictive corner radii. To overcome the physical constraints of material removal intrinsic to confined deep-cavity environments, a high-intensity, five-pass sequential polishing regimen was implemented.

Upon completion of the respective polishing protocols for both the convex and concave features, 3D surface topographies and quantitative roughness metrics were comprehensively evaluated utilizing a 3D optical profilometer (VR-5000, Keyence, Osaka, Japan). The measurements were executed in high-magnification mode. To ensure the absolute fidelity of the extracted topographical features and avoid artificial data smoothing, both the S-filter and L-filter were intentionally disabled during the evaluation. A standard planar tilt correction was applied solely to level the measurement baseline. Strictly conforming to the ISO 25178 standard [6] for areal surface texture measurement, the evaluation was conducted under standardized conditions to ensure data reliability and comparability.

## 3. Results and Discussion

### 3.1. Mechanical Response of the PA66/Diamond Composite

As summarized in Table 2, mechanical characterization reveals that the FGF-fabricated PA66/diamond composite demonstrates exceptional structural robustness under mechanical loading. Descriptive statistical analysis (specifically calculating the mean, standard deviation, and coefficient of variation) across the six replicate specimens yielded a mean ultimate tensile strength (UTS) of 59.39 ± 15.41 MPa, alongside an average apparent elastic modulus of 471.20 MPa and a mean yield strength of 55.18 MPa. Notably, the peak-performing specimen attained a UTS of 78.90 MPa—correlating with a maximum tensile load of 795.33 N—and a peak yield strength of 70.34 MPa. Regarding material ductility and toughness, the composite exhibited an average elongation at break of 27.42 ± 7.41% (peaking at 36.92%, which equates to a crosshead displacement of 9.23 mm at failure). The relatively high coefficient of variation (CV) in the tensile properties (specifically 25.94% for UTS and 27.03% for elongation at break) stems from the inherent microstructural heterogeneity of highly filled 3D-printed composites. Specifically, random agglomerations of the 25 wt% diamond micro-particles act as severe stress concentrators under tension. Additionally, the layer-by-layer FGF process inherently generates stochastic inter-bead micro-voids. The unpredictable spatial distribution of these internal defects creates random crack initiation sites, precipitating premature localized fractures in weaker specimens (e.g., Specimen 5, which exhibited a minimum UTS of 36.09 MPa) and driving the statistical dispersion observed in the macroscopic mechanical responses. These empirical findings substantiate that, despite incorporating a 25 wt% mass fraction of superhard diamond abrasives, the PA66 matrix retains superior structural integrity and substantial fracture toughness. Consequently, the formulated composite definitively satisfies the rigorous tribo-mechanical prerequisites for high-load abrasive machining applications.

At the locus of physical and chemical interfacial interlocking, the macromolecular architecture of PA66 comprises alternating highly polar amide linkages and flexible methylene segments. While explicit spectroscopic characterization (e.g., FTIR or XPS) was not conducted in this study to quantify the chemical interactions, it is rationally deduced that this physicochemical interfacial interlocking potentially facilitated the formation of a dense intracatenary hydrogen-bonded network within the polymer matrix. Concurrently, the surfaces of diamond micro-abrasives spontaneously oxidize under ambient conditions, generating oxygen-rich functional groups such as hydroxyl and carboxyl species. Synergistically activated by the intense thermomechanical shear fields intrinsic to FGF melt extrusion, the highly polar amide groups of PA66 engage in profound dipole–dipole interactions—and potentially interfacial hydrogen bonding—with these oxygenated diamond surfaces [7]. This physicochemical synergy promotes exemplary wetting and encapsulation of the diamond particulates by the polymer melt, thereby precipitating a robust interphase [8,9].

Upon the application of external tensile or shear loading, this resilient interfacial adhesion effectively circumvents the premature debonding of the superhard reinforcing phase. From a fracture mechanics perspective, as microcracks nucleate and propagate through the polymeric matrix, their interaction with the rigid diamond particulates is highly likely to induce crack tip deflection, pinning, or bifurcation [10]. These complex energy-dissipation mechanisms substantially consume external strain energy while efficiently transferring stress across the interface to the high-modulus diamond skeleton. In this load-bearing architecture, the diamond abrasives function as “rigid cross-linking nodes” within the macromolecular network, whereas the encompassing flexible methylene segments provide the necessary free volume for stress-induced conformational transitions. This synergistic deformation mechanism thoroughly elucidates the microstructural origins of the concurrent high yield strength and exceptional elongation at break previously documented in Table 2 [11,12].

Although specific crystallographic evaluations, such as X-ray diffraction (XRD), were not conducted to directly quantify macromolecular alignment, the high shear rates experienced by the semi-crystalline PA66 melt during nozzle extrusion are generally understood to induce a degree of flow-induced molecular orientation. Consequently, while the exact microstructural anisotropy remains theoretically inferred rather than empirically verified in this specific investigation, this extrusion-driven alignment phenomenon is hypothesized to contribute to the observed macroscopic mechanical directionality, occasionally interacting with internal defect distributions to influence the global variance in tensile responses [13]. Consequently, the specific macroscopic geometric design facilitates the required passive structural compliance. Rather than possessing localized customized mechanical properties, this macroscopic structural configuration—comprising the radial gaps and the hyperelastic buffer—enables the composite tool to geometrically adapt to the high-curvature transitions of the mold cavity, ensuring uniform contact pressure without requiring complex dynamic force feedback [14].

To contextualize the industrial viability of the synthesized composite, its mechanical robustness was benchmarked against conventional compliant polishing materials. To contextualize the industrial viability of the synthesized composite, its mechanical robustness was qualitatively benchmarked against conventional compliant polishing materials. Unlike traditional wool bobs or cast polyurethane pads, which frequently suffer from rapid abrasive depletion and severe structural yielding under heavy compressive loads, the FGF-printed PA66 matrix exhibits superior shear resistance. However, it is important to clearly state that in the absence of direct quantitative benchmarking (such as comparative material removal rates or volumetric wear measurements under identical kinematic conditions), this comparison remains a qualitative assessment. While the documented tensile properties support sustained abrasive retention, conclusions regarding its total industrial durability relative to existing tools must be drawn with caution.

### 3.2. Topographical Evolution of Convex Surfaces During Adaptive Polishing

**Figure 4:**
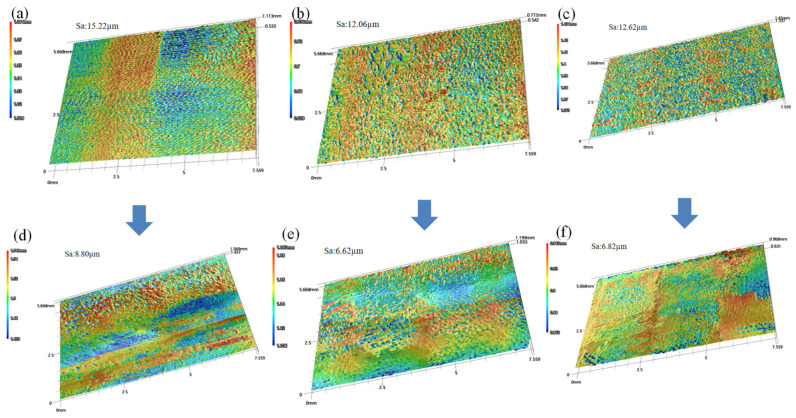
3D profilometric scans illustrating the topographical evolution of the convex mold surface: (**a**–**c**) as-milled topography prior to polishing; (**d**–**f**) final surface topography following the adaptive polishing regimen.

The complex surface polishing experiments were designed to validate the conformal planarization capabilities of the elastic composite tool on non-planar topographical features. Prior to the polishing intervention, the as-milled P20 mold steel substrate exhibited a characteristically dull macroscopic appearance, plagued by a high initial average surface roughness of 13.33 µm. Profilometric scans acquired from three distinct evaluation zones (the anterior, medial, and posterior regions of the mouse mold contour) These specific locations were strategically selected to represent distinct characteristic topographical features with varying localized curvatures across the complex freeform surface. This approach ensures a comprehensive and representative assessment of the global surface condition rather than relying on a single, arbitrary coordinate. The initial evaluation of these representative zones revealed a topography dominated by dense, terrace-like mechanical tool marks and distinct regions of non-uniform localized stress (Figure 4a–c).

From a metallurgical perspective, AISI P20 mold steel is known in established literature to possess a heterogeneous, multiphase microstructure due to bulk quenching and tempering cycles [15]. During conventional rigid polishing, this inherent microstructural heterogeneity often acts as the primary catalyst for surface degradation. Rigid tools, lacking localized elastic yielding, struggle to uniformly process the alternating softer matrix and ultra-hard carbide precipitates, frequently leading to localized pitting or abrasive pull-out. Macroscopically, this erratic micro-cutting behavior translates into “orange peel” defects and deep scratches [16,17]. By recognizing this established metallurgical context, the necessity for a compliant intervention becomes evident; the composite elastic polishing tool is specifically designed to dynamically buffer against these hard phases, fundamentally preventing the macroscopic rigid-collision defects typically observed on P20 substrates.

**Figure 5:**
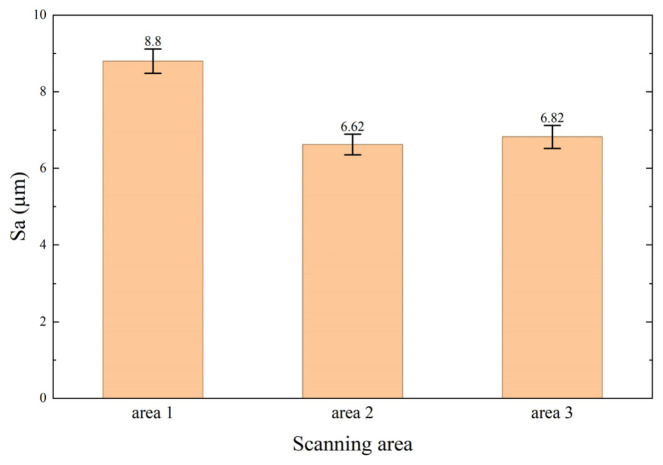
$S_{a}$ bar chart of the three polished regions, with error bars indicating the respective $S_{a}$ ranges.

The topographical evolution of the convex surface exhibited a distinct, step-wise planarization trajectory. Following the initial three continuous polishing passes, the macroscopic surface morphology demonstrated noticeable refinement. However, constrained by the fixed kinematic posture inherent to three-axis machining, the tool-workpiece interaction remained suboptimal across the steep marginal geometries. Consequently, these peripheral “blind zones” retained severe vestiges of the original rough milling defects. The subsequent implementation of the customized segmented regional polishing strategy during the fourth pass profoundly rectified this limitation, significantly enhancing abrasive coverage within the marginal zones and instigating a global reduction in surface roughness. Upon the completion of the fifth and final intensive reciprocating pass, residual milling striations were virtually eradicated. The convex apex and associated low-gradient topologies achieved a highly uniform and smooth finish, corroborating a substantial reduction in the surface roughness to a statistical average of 7.42 µm across the three evaluation zones. This progressive topographical amelioration is quantitatively and visually documented in the post-polishing profilometric scans (Figure 4e–f), evaluated across the identical spatially correlated measurement zones established prior to machining [18]. Concurrently, a complementary bar chart summarizing these Sa values is provided, wherein statistical error bars (Figure 5) are explicitly incorporated to delineate the global topographical variance across the scanned regions.

When the polymeric polishing head, characterized by a specific elastic modulus, contacts the convex P20 steel surface under the normal pressure of the CNC spindle, the tool tip undergoes macroscopic elastic deformation. This geometric adaptation transitions the tool-workpiece interface from a theoretical “point” or “line” contact into a spatially extended “compliant polishing footprint” [19].

Within this compliant contact zone, the localized normal stress distribution is fundamentally governed by the relative height of the microscopic surface asperities. Upon encountering initial milling peaks—measuring up to 13.33 µm—the PA66 matrix undergoes local compression. This structural response effectively concentrates the contact load at the peaks, forcing the embedded diamond micro-abrasives to engage the metallic surface, thereby executing efficient micro-cutting. Conversely, within the topographical valleys, the passive compliance of the elastic matrix minimizes penetration, leading to a substantial reduction in the localized contact pressure. This preferential contact mechanism ensures that the diamond particles are predominantly active at the high-frequency asperities while exhibiting negligible material removal within the valleys, facilitating a controlled surface planarization [20].

Crucially, at the micro-scale, the interaction is hypothesized to be governed by a dynamic “yielding mechanism.” When the diamond grains, operating at high rotational velocities, impinge upon the exceptionally hard carbide precipitates inherent to P20 steel, the instantaneous cutting resistance surges precipitously. Dictated by Newtonian reaction forces, the diamond particles are thrust back against the supporting PA66 matrix. Leveraging the matrix’s superior elongation at break and underlying elasticity, the macromolecular chains undergo transient compression. This compliance permits the diamond grains to temporarily “retreat” into the bulk polymer, thereby effectively avoids catastrophic, rigid collisions. Once the abrasive traverses the hard phase, the elastic recovery force of the polymer network propels the diamond outward to resume steady-state cutting. This proposed self-adaptive yielding mechanism is considered to play a key role in minimizing the formation of deep gouges and “orange peel” defects typically induced by grain fragmentation or forced abrasive dragging.

As the macro-scale milling striations are progressively sheared away by the diamond micro-edges, the machining process transitions strictly into the ductile-regime cutting phase. Because the inherent compliance of the polishing head drastically attenuates the maximum undeformed chip thickness for individual abrasive grains, the depth of cut is rigorously confined within the micro-plastic deformation threshold of the P20 steel, effectively precluding brittle fracture and micro-crack initiation. Following three, and ultimately five, polishing iterations, the initially dull and rough convex surface transitions into a smooth, completely obliterating the original “terrace-like” topography. Evaluated through the progressive topographical evolution, this phase marks a fundamental physical reconstitution of the near-surface macro-morphology within the ductile deformation regime. Consequently, as the number of polishing cycles increases, both high- and mid-frequency topographical errors are rigorously suppressed, culminating in a qualitative leap in the global surface finish [16]. To indirectly support this proposed dynamic yielding mechanism in the absence of in situ microstructural observation, macroscopic and mesoscopic evaluations of the post-polishing tool interface, coupled with the intrinsic mechanical properties of the composite, were comprehensively analyzed. Optical inspections of the functional abrasive layer following five high-intensity polishing cycles revealed no evidence of catastrophic matrix tearing, thermal degradation, or widespread abrasive pull-out. The sustained retention of the diamond particulates physically corroborates the matrix’s structural resilience. Furthermore, this resilience is fundamentally supported by the high elongation at break (27.42%) established in the tensile characterizations (Section 3.1), which provides the requisite macromolecular free volume for transient elastic buffering. Crucially, the complete absence of deep dragging gouges or brittle fracture marks on the final P20 steel topography (as confirmed by 3D profilometry) provides qualitative support for this proposed mechanism. This topographical state suggests that the abrasive grains engaged in a steady-state, micro-ductile cutting action rather than rigid plowing, thereby verifying that transient impact forces were effectively absorbed and dissipated by the localized structural yielding of the PA66 network.

### 3.3. Topographical Evolution of Concave Surfaces During Adaptive Polishing

**Figure 6:**
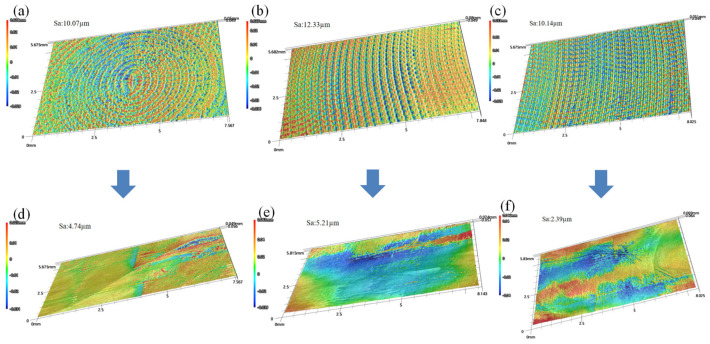
3D profilometric scans illustrating the topographical evolution of the concave mold surface: (**a**–**c**) as-milled topography prior to polishing; (**d**–**f**) final surface topography following the adaptive polishing regimen.

Within the deep concave cavities, the as-milled topography exhibited a severe initial average surface roughness of 10.84 µm (Figure 6a–c). Following the initial three baseline polishing iterations, machining uniformity proved substantially inadequate. From the perspective of multi-axis CNC kinematics and robotic polishing theory, this deficiency originates from the inherent conflict between spatial tool-axis vector singularities and geometric curvature mismatch [21]. When a standard flexible tool descends with a fixed, vertical posture into a high-steepness, small-radius concave feature, volumetric interference dictates that the tool’s periphery prematurely collides with the cavity sidewall. To circumvent physical machine overload, standard CNC obstacle-avoidance algorithms forcibly retract the *Z*-axis. Consequently, the tool tip is structurally “bridged” over the transitional corner radii. In the context of polishing dynamics, this phenomenon drastically truncates the effective compliant footprint, dropping normal contact pressure to zero and entirely neutralizing the material removal mechanism in these restrictive “dead zones,” thereby preserving deep, original milling marks [22,23].

To mathematically and kinematically resolve this high-dimensional dynamic control issue regarding the polishing footprint, the CNC compensation trajectories and engagement angles were comprehensively optimized for the fourth pass. Aligning with contemporary advances in freeform surface path planning, continuous interpolation of lead and tilt angles was introduced into the control system [24]. Through multi-axis coordinated rotation, the tool engagement paradigm shifted: rather than machining via the polar apex, the non-polar spherical regions of the tool were maneuvered to actively interface with the blind zones. This strategic kinematic posture adjustment elegantly bypasses the physical interference of the tool shank and spindle, accurately guiding the compressive deformation zone of the flexible PA66 matrix directly into the previously inaccessible asperities.

**Figure 7:**
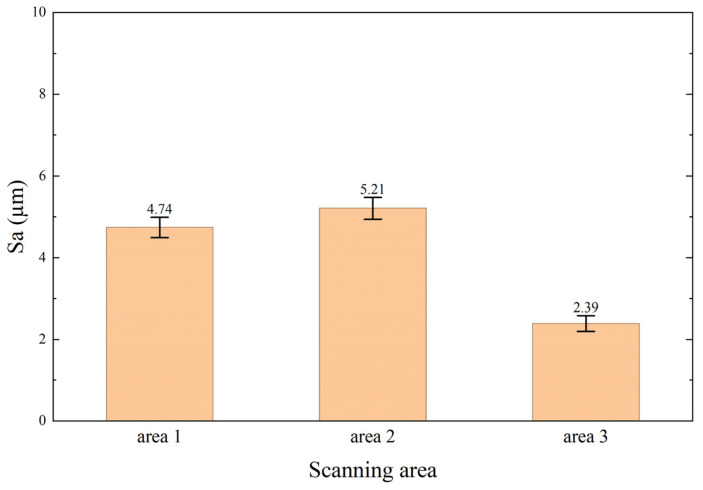
$S_{a}$ bar chart of the three polished regions, with error bars indicating the respective $S_{a}$ ranges.

The implementation of this optimized strategy yielded a drastically expanded effective cutting envelope, successfully eradicating the marginal blind zones and prompting a steady ascension in surface finish. Upon the execution of a fifth, high-intensity sequential polishing pass, the surface topography of the entire concave structure was dramatically planarized, approaching a near-defect-free, exceptionally smooth level. While infinitesimal machining flow marks persisted in the most extreme, highly localized corner extremities, the global surface achieved a definitive transformation from a rough-milled topography to a substantially refined and smooth substrate. Evaluated across the three spatially correlated measurement zones (anterior, medial, and posterior), statistical analysis revealed that the final surface roughness plummeted to an average of 4.11 µm, as corroborated by the post-polishing profilometric scans (Figure 6d–f). These quantified Sa distributions are comprehensively compiled in the corresponding bar chart (Figure 7), wherein explicit statistical error bars delineate the localized topographical variation within each respective zone. This low variance across distinct spatial coordinates demonstrates the high uniformity of material removal achieved within the complex concave topography.

Concurrently, this kinematics-based adaptive compensation drastically broadens the geometric adaptability of the polishing process. The inherent viscoelasticity of the FGF-fabricated composite effectively dampens minute normal overcut errors—typically induced by servo lag or rotary axis backlash during five-axis simultaneous interpolation. In this context, the elastic tool functions as a “mechanical low-pass filter” at the interface between transient machine tool dynamics and the workpiece, thereby guaranteeing the smooth attenuation of residual tool marks.

Fundamentally, the exceptional polishing uniformity and the resulting transition toward an ultra-smooth surface topography achieved on the concave surface transcend mere kinematic correction; they are intrinsically governed by the precise spatiotemporal distribution of the material removal rate across variable-curvature topographies. The classical Preston equation (Equation (1)) mathematically delineates this linear material removal phenomenon:

(1) $$MRR=\frac{dh}{dt}=k\cdot P\cdot V$$

Among these, h denotes the material removal depth, k is the empirical Preston coefficient, P represents the normal contact pressure, and V defines the relative sliding velocity.

However, the classical linear Preston hypothesis becomes fundamentally invalid when applied to non-planar, deep-cavity topographies characterized by variable curvatures. As the local curvature gradient of the concave surface dynamically fluctuates, the morphological footprint of the compliant polishing tool is subjected to severe nonlinear distortion. Consequently, the transient contact stress at any arbitrary coordinate within the engagement zone becomes exceedingly complex to analytically resolve. To accurately model this dynamic material removal process, an extended, generalized Preston integral model (Equation (2)) has been established in contemporary literature:

(2) $$h\left(x,y\right)=\int_{0}^{T}k\left(P,V,\mu \right)\cdot P\left(x,y,t\right)\cdot V\left(x,y,t\right)dt$$

During toolpath optimization, the CNC architecture must execute iterative refinements of the tool dwell time concurrently with spatial posture adjustments. To rectify the inadequate material removal rates observed in restrictive corner radii, restoring the normal contact pressure via tool-axis tilt compensation is, in itself, insufficient. It is imperative to strategically decelerate the machine tool’s feed rate within these high-curvature, topographically complex zones, thereby substantially increasing the tool dwell time. Conversely, across the low-gradient central basin, the feed kinematics are purposefully accelerated to preclude localized over-polishing [25].

The sustained operational integrity of the polishing head throughout five high-intensity, variable-load, and variable-posture machining iterations—governed by the aforementioned generalized Preston dynamics—is fundamentally attributed to the prolonged fatigue resistance inherent to the high-strength and highly ductile PA66 composite matrix. Strikingly, the tool exhibited no macroscopic fracture or catastrophic spalling induced by cyclic stress concentrations; instead, it delivered a continuous, highly stable micro-cutting force output. Ultimately, while infinitesimal flow marks persisted within highly localized microscopic boundaries, the adaptive process achieved a definitive, cross-scale physical topographical reconstitution, transforming the original rough 3D-milled morphology into a substantially refined and smooth substrate.

### 3.4. Evaluation of Tool Durability and Machining Consistency

To evaluate the durability and wear characteristics of the 3D-printed compliant tool, the morphological integrity and machining consistency were comprehensively analyzed over the multi-pass polishing regimen. Optical and macroscopic inspections of the composite tool following the five high-intensity sequential polishing cycles revealed structural robustness; neither macroscopic petal fracture nor interfacial delamination between the TPU buffer and the PA66 active layer was observed. Furthermore, no massive abrasive pull-out or catastrophic thermal degradation of the polymer matrix occurred.

Crucially, the machining consistency is quantitatively corroborated by the topographical evolution of the mold steel. During the continuous polishing cycles, the surface roughness ($S_{a}$) exhibited a steady, monotonic decline (reaching 7.42 µm on convex surfaces and 4.11 µm in concave cavities). If severe tool wear, abrasive blunting, or significant abrasive loss had transpired, the material removal rate (MRR) would have plummeted, leading to a stagnation or deterioration in surface finish. The continuous amelioration of the global surface topography suggests that the tool maintains stable micro-cutting performance within the limited testing window. However, it must be clearly acknowledged that the current experimental scope is restricted to five sequential polishing passes. While these initial observations are promising, the statistical robustness regarding repeatability and long-term industrial tool life cannot be definitively established from this specific experimental design and require further extended evaluation. This observed initial durability is fundamentally attributed to the high ultimate tensile strength (average UTS: 59.39 MPa) and the previously discussed theoretical macro-micro yielding mechanism of the composite matrix, which securely anchors the diamond particulates while dynamically absorbing severe tribological impacts.

## 4. Conclusions

This study introduces a novel automated polishing paradigm predicated on a “passive compliance” mechanism. Utilizing fused granulation fabrication (FGF) additive manufacturing, a monolithic, five-petal multi-material composite elastic polishing tool was successfully engineered. This research systematically investigates the planarization mechanisms driving the transition from brittle fracture to microscopic ductile-regime cutting under severe operational conditions. Furthermore, multi-pass adaptive machining evolution experiments were executed on complex non-planar mold geometries. The principal conclusions are summarized as follows:

(1) A five-petal composite elastic polishing tool, structurally integrating a passive TPU compliant buffer layer with an active PA66/diamond micro-cutting functional layer, was successfully fabricated. Mechanical characterization revealed a maximum ultimate tensile strength of 78.90 MPa and an elongation at break of 27.42%. These metrics demonstrate exceptional fracture toughness and structural robustness under heavy-load polishing conditions, providing a structural approach to mitigate the pervasive engineering challenge of premature abrasive debonding inherent to conventional finishing processes.
(2) Facilitated by the macroscopic elastic deformation and a proposed micro-yielding mechanism, the developed tool fundamentally transitions the material removal paradigm from forced, rigid mechanical scratching to a precision, damage-free “differential asperity planarization” mode. Empirical validation on convex P20 mold steel topographies demonstrated the effective suppression of micro-crack initiation and deep, rigid-collision-induced scratches. Following the polishing intervention, the original macroscopic milling striations were largely planarized, yielding a significant reduction in average surface roughness from 13.33 µm to 7.42 µm, thereby realizing a transformative enhancement in global surface quality.
(3) To overcome the kinematic interference bottlenecks endemic to deep concave cavities with variable curvatures, the five-petal structural design—synergized with the “mechanical low-pass filtering” effect of the TPU substrate—demonstrated the capacity to passively and autonomously absorb spatial interpolation errors. Integrated with a segmented, dynamically compensated CNC toolpath strategy, the average surface roughness of the deep concave features was reduced from 10.84 µm to 4.11 µm. The localized topography at the restrictive sidewall junctions achieved a highly consistent and smooth surface finish without macroscopic defects. This technological framework effectively circumvents the traditional reliance on capital-intensive, six-degree-of-freedom (6-DOF) robotic platforms for the precision finishing of complex freeform surfaces.

Future research will focus on investigating the long-term tribological wear mechanisms of the 3D-printed composite matrix under extended industrial life cycles, as well as exploring the integration of in situ acoustic emission or force-feedback monitoring to further optimize the dynamic polishing footprint on ultra-complex optical freeform surfaces.

## Figures and Tables

**Table 1:** Measured dimensional data of the tensile test specimens.

Specimen No.	Test Duration, t (s)	Width, w (mm)	Thickness, h (mm)	Original Gauge Length, L0 (mm)	Extensometer Gauge Length, Le (mm)
1	29.3	4.2	2.4	25	12
2	29.5	3.9	3	25	12
3	27.3	3.6	2.56	25	12
4	30.6	4.29	2.71	25	12
5	17.6	4.25	2.86	25	12
6	23	3.6	2.88	25	12
Max	30.6	4.29	3	25	12
Min	17.6	3.6	2.4	25	12
Mean	26.22	3.97	2.74	25	12
SD	4.57	0.29	0.20	0	0
CV (%)	17.43	7.35	7.47	0	0

**Table 2:** Tensile test data.

Specimen No.	Elongation at Break, ϵb (%)	Ultimate Tensile Strength, $\sigma_{UTS}$ (Mpa)	Max Tensile Load, Fmax (N)	Max Displacement, ΔLmax (mm)	Elastic Modulus, E (MPa)	Yield Strength, σy (MPa)
1	21.04	78.90	795.33	5.26	557.30	70.34
2	36.03	63.41	741.91	9.01	448.33	57.96
3	27.86	70.46	649.34	6.96	649.71	69.69
4	36.92	65.97	766.97	9.23	469.14	59.88
5	16.32	36.09	438.72	4.08	306.31	32.35
6	26.38	41.52	430.52	6.59	396.44	40.87
Max	36.92	78.90	795.33	9.23	649.71	70.34
Min	16.32	36.09	430.52	4.08	306.31	32.35
Mean	27.42	59.39	637.13	6.86	471.20	55.18
SD	7.41	15.41	150.04	1.85	109.99	14.12
CV (%)	27.03	25.94	23.55	27.03	23.34	25.59

## Data Availability

The original contributions presented in this study are included in the article. Further inquiries can be directed to the corresponding authors.
